# Species distribution and *in vitro *antifungal susceptibility of oral yeast isolates from Tanzanian HIV-infected patients with primary and recurrent oropharyngeal candidiasis

**DOI:** 10.1186/1471-2180-8-135

**Published:** 2008-08-12

**Authors:** Omar JM Hamza, Mecky IN Matee, Mainen J Moshi, Elison NM Simon, Ferdinand Mugusi, Frans HM Mikx, Wim H van Palenstein Helderman, Antonius JMM Rijs, André JAM van der Ven, Paul E Verweij

**Affiliations:** 1 Department of Oral Surgery and Oral Pathology, Muhimbili University of Health and Allied Sciences, Dar es Salaam, Tanzania; 2 Department of Microbiology and Immunology, Muhimbili University of Health and Allied Sciences, Dar es Salaam, Tanzania; 3 Department of Biological and Preclinical studies, Institute of Traditional Medicine, Muhimbili University of Health and Allied Sciences, Dar es Salaam, Tanzania; 4 Department of Internal Medicine, Muhimbili University of Health and Allied Sciences, Dar es Salaam, Tanzania; 5 WHO Collaborating Center, Dentistry, Radboud University Nijmegen Medical Center, Nijmegen, The Netherlands; 6 Department of Medical Microbiology, Radboud University Nijmegen Medical Center, Nijmegen, The Netherlands; 7 Nijmegen University Center for Infectious Diseases, Nijmegen, The Netherlands; 8 Department of General Internal Medicine, Radboud University Nijmegen Medical Center, Nijmegen, The Netherlands

## Abstract

**Background:**

In Tanzania, little is known on the species distribution and antifungal susceptibility profiles of yeast isolates from HIV-infected patients with primary and recurrent oropharyngeal candidiasis.

**Methods:**

A total of 296 clinical oral yeasts were isolated from 292 HIV-infected patients with oropharyngeal candidiasis at the Muhimbili National Hospital, Dar es Salaam, Tanzania. Identification of the yeasts was performed using standard phenotypic methods. Antifungal susceptibility to fluconazole, itraconazole, miconazole, clotrimazole, amphotericin B and nystatin was assessed using a broth microdilution format according to the guidelines of the Clinical and Laboratory Standard Institute (CLSI; M27-A2).

**Results:**

*Candida albicans *was the most frequently isolated species from 250 (84.5%) patients followed by *C. glabrata *from 20 (6.8%) patients, and *C. krusei *from 10 (3.4%) patients. There was no observed significant difference in species distribution between patients with primary and recurrent oropharyngeal candidiasis, but isolates cultured from patients previously treated were significantly less susceptible to the azole compounds compared to those cultured from antifungal naïve patients.

**Conclusion:**

*C. albicans *was the most frequently isolated species from patients with oropharyngeal candidiasis. Oral yeast isolates from Tanzania had high level susceptibility to the antifungal agents tested. Recurrent oropharyngeal candidiasis and previous antifungal therapy significantly correlated with reduced susceptibility to azoles antifungal agents.

## Background

Oropharyngeal candidiasis (OPC) is the most frequent opportunistic infection encountered in human immunodeficiency virus (HIV)-infected individuals [1-4]. It occurs in up to 90% at some point during the course of HIV disease [1,3,4]. The occurrence of OPC is associated with CD4 T-lymphocyte below 200 cells/mm^3^, high viral loads and disease progression [2,5,6]. The prolonged course of HIV infection predisposes these patients to recurrent episodes of OPC that can increase in frequency and severity with progressive HIV disease [7]. The advent of highly active antiretroviral therapy (HAART) has permitted suppression of viral replication and a partial recovery of CD4 T-lymphocyte count in HIV infected patients [8]. Although the incidence and prevalence of opportunistic infections have been reduced worldwide due to use of HAART [9-12], OPC remains the most frequent HIV-associated oral lesion in most developing countries, including Tanzania [6].

The prolonged management of OPC might cause the development of drug-resistant OPC and there have been reports of emergence of resistance to antifungal agents in HIV/AIDS patients with OPC [13,14]. OPC due to resistant *Candida *isolates fails to respond to antifungal treatment with appropriate doses for a standard duration of time [15,16]. The other possibility is that, repeated exposure to antifungal agents may predispose to a shift to non-albicans *Candida *species and associated refractory and recurrent infections [17-20]. However, a surveillance study done in the United Kingdom demonstrated that there was little difference in antifungal susceptibilities of *Candida *species isolated from immunocompetent patients who had a history of previous antifungal therapy compared with those who had not received antifungal treatment [21].

OPC increases morbidity and mortality and reduces length and quality of life of HIV/AIDS patients, and therefore requires prompt diagnosis and adequate therapy. In vitro susceptibility testing is clinically useful in predicting which patients are likely to respond to therapy [22]. In Tanzania, like many other developing countries, in vitro antifungal testing is not performed routinely [23,24]. Therefore, little is known regarding the in vitro antifungal susceptibility of *Candida *species isolated from HIV-infected patients with OPC in Tanzania.

The aim of our study was to determine the species distribution of *Candida *isolates obtained from Tanzanian HIV-infected patients with primary and recurrent OPC. For each isolate the susceptibility profiles of six antifungal agents was determined and compared for treatment naive patients and repeatedly antifungal exposed patients.

## Methods

### Patients and setting

Participants were recruited at the Muhimbili National Hospital (MNH) HIV clinic in Dar es Salaam, Tanzania from January 2006 to September 2007. Patients were eligible for the study if they were HIV-positive, 18 years of age and above with clinical picture of OPC, characterized by creamy, white, curd like patches, or by typical erythematous lesions on the oral or/and pharyngeal mucosa. Patients were included after obtaining their written informed consent. Patients were interviewed using a standard structured questionnaire and their hospital records were also used to determine the previous episodes of OPC, use of antifungal agents and past medical conditions. A standard oral examination method recommended by WHO was used [25]. Blood was collected in EDTA tubes from each patient for enumeration of CD4+ and CD8+ T cells using a fluorescent activated cell sorter (FACS) count machine after staining patients' blood with monoclonal antibodies [26]. Primary OPC was defined as the one that occur for the first time and recurrent OPC as the second episode and above during the course of HIV disease.

### Oral isolates and species identification

Specimens were obtained by firmly swabbing the lesion site with sterile cotton wool swab [27]. The swabs were cultured on sabouraud dextrose agar (SDA) (Oxoid Ltd, Hampshire, England) supplemented with 0.02% chloramphenicol and incubated aerobically at 30°C for 5 days. Isolates were identified to species level by colonial morphology on SDA and microscopic morphology. The germ tube production test was done. To distinguish between *C. albicans *and *C. dubliniensis*; all germ tube positive isolates were subcultured on SDA and incubated at 45°C aerobically and an isolate which did not grow at 45°C, was identified using AUXACOLOR 2 (Bio-Rad, France). Germ tube negative yeasts were identified using AUXACOLOR 2 (Bio-Rad, France) and the auxanographic method [28]. In the presence of multiple colony morphologies, all were identified to species level. After the final identification, isolates were stored at -80°C in 50% glycerol until susceptibility tests were performed.

### In vitro antifungal susceptibility testing

The *in vitro *activities of fluconazole (Pfizer, Kent, UK), itraconazole, miconazole (Janssen-Pharmaceutica N.V., Beerse, Belgium), clotrimazole (Sigma Aldrich, Steinheim, Germany), amphotericin B (Bristol-Myers Squibb, Woerden, The Netherlands) and nystatin (Sigma Aldrich, Steinheim, Germany) were assessed. The minimum inhibitory concentration (MIC) was determined using a broth microdilution format (M27-A2) according to the guidelines of the Clinical and Laboratory Standard Institute (CLSI) [29], and all the testing done in duplicates. The respective manufacturers provided the antifungal agents as standard powders. Fluconazole was dissolved in sterile distilled water and itraconazole, miconazole, clotrimazole, amphotericin B and nystatin were dissolved in dimethyl sulfoxide (DMSO) to make stock solutions. RPMI 1640 medium with L-glutamine, without sodium bicarbonate (GIBCO BRL, Life Technologies, Woerden, The Netherlands) and buffered with morpholinepropanesulfonic acid (MOPS) at 0.165 M (Sigma-Aldrich Chemie GmbH, Steinheim, Germany) was used as a test medium. Serial twofold dilutions and final dilutions of each antifungal agent were prepared in RPMI 1640 medium [29]. The range of fluconazole concentrations tested was 0.063–64 *μ*g/ml and for itraconazole, miconazole, clotrimazole, amphotericin B and nystatin the range was 0.016–16 *μ*g/ml. Aliquots of 100 *μ*l of each antifungal agent at a concentration two times the targeted final concentration were dispensed in the wells of flat bottom 96-well microtiter plates (Costar, Corning, N.Y). Prior to testing, isolates were subcultured on SDA at 30°C under aerobic conditions for 24 hours. Yeast inocula were prepared spectrophotometrically and further diluted in RPMI 1640 medium in order to obtain a final concentration of 0.5 to 2.5 × 10^3 ^CFU/ml [29]. A constant volume (100 *μ*l) of the innoculum was added to each microdilution well containing 100 *μ*l of the serial dilution of the antifungal agents to reach final concentrations. The microtiter plates were incubated at 35°C for 48 hours.

The minimum inhibitory concentrations (MICs) were determined after 48 hours, after which readings were performed spectrophotometrically with a microplate reader (Anthos ht III; Anthos Labtec Instruments, Salzburg, Austria) at 405 nm using modified broth microdilution format (M27-A2) as described previously [21,30]. The optical densities (ODs) of the blanks were subtracted from the ODs of the inoculated plates and the percentages of growth for each well were calculated. The MICs of polyene antifungals (amphotericin B and nystatin) were defined as the lowest concentrations that inhibit growth by 100%. The MICs of the azoles (fluconazole, itraconazole, clotrimazole and miconazole) were defined as the lowest concentrations that inhibit growth by 50%. Antifungal activity was expressed as the MIC of each isolate to the antifungal agent. The MIC values were the average of MICs of the first and duplicate measurement. The MIC values for fluconazole and itraconazole were compared to the CLSI interpretative guidelines on antifungal susceptibility testing. MICs of ≤ 8 *μ*g/ml considered as susceptible, 16 – 32 *μ*g/ml as susceptible dose dependent (SSD) and ≥ 64 *μ*g/ml as resistant for fluconazole and for itraconazole, ≤ 0.125 *μ*g/ml as susceptible, 0.25 – 0.5 *μ*g/ml as SDD and ≥ 1 *μ*g/ml as resistant [29]. American Type Culture Collection (ATCC) strains recommended by CLSI, *C. parapsilosis *(ATCC 22019) and *C. krusei *(ATCC 6258) were used as controls for each test.

### Ethical issues

The Ethics Committees of the Muhimbili University of Health and Allied Sciences (MUHAS) and the Muhimbili National Hospital, Dar es salaam, Tanzania approved the study. All patient information and test results were confidentially kept.

### Data and statistical analysis

The geometric mean of MICs, MIC_50 _and MIC_90 _were calculated. The MIC_50 _and MIC_90 _values were calculated as the concentrations of antifungals that were able to inhibit 50% and 90% of the isolates, respectively. The high and low off-scale MICs were included in the analysis by conversion to the next higher and lower drug concentrations, respectively. An isolate was considered resistant to fluconazole and itraconazole if average MICs were greater or equal to their respective CLSI breakpoints [29]. Data were analyzed using the SPSS version 14.0. Antifungal susceptibility of isolates from patients with primary OPC was compared with those of isolates from patients with recurrent OPC. Similarly, the antifungal susceptibility of isolates from patients who had not received an antifungal agent was compared with those of isolates from patients who had previously received antifungal therapy. Comparison of species distribution and antifungal susceptibility rates was performed using Fisher's exact test and Mann-Whitney *U-*test. A *P*-value of < 0.05 was considered significant.

## Results

### Patients' characteristics

Two hundred and ninety two (292) HIV infected patients with OPC, 164 with primary OPC and 128 with recurrent OPC, participated in the study. Pseudomembranous candidiasis was the most predominant type of OPC 194 (66.4%) followed by combinations of pseudomembranous and erythematous 66 (22.6%), pseudomembranous and angular cheilitis 12 (4.1%), pseudomembranous and hyperplastic candidiasis 9 (3.1%), hyperplastic candidiasis 7 (2.4%) and erythematous candidiasis were the least 4 (1.4%). Patients' ages ranged from 18 to 75 years, median 34 years of whom 218 (74.7%) were females and 74 (25.3%) were males. The majority of the patients with recurrent OPC had a history of one previous episode of OPC (n = 92, 71.8%) and the range of recurrences was 1 to 4 episodes. Their CD4+ T cells values ranged from 1 to 699 cells/mm^3 ^with a median count of 93 cells/mm^3 ^and 229 patients (78.4%) had CD4+ T cells count below 200 cells/mm^3^. One hundred and seven patients (36.6%) were under HAART, predominantly (72.9%) a combination of stavudine, lamivudine and nevirapine and 65 patients (60.7%) were on HAART for duration below six months.

### Distribution of isolates

Two hundred and ninety six (296) clinical oral yeasts were isolated from the 292 patients with OPC. One hundred and sixty five (165) isolates were from patients with primary OPC and 131 isolates from patients with recurrent OPC. The species distribution as presented in Table 1 show that *C. albicans *was the most frequently isolated species (84.5%) followed by *C. glabrata *(6.8%) and *C. krusei *(3.4%). *Saccharomyces cerevisiae *was the only isolated non-*Candida *species from these patients. Four patients had mixed infection, two harbored *C. albicans *and *C. krusei *and two other patients had *C. glabrata *and *C. krusei*. Among four patients with mixed infection, three patients had recurrent OPC and one patient who harbored *C. glabrata *and *C. krusei *had primary OPC. There was no statistically significant difference in species distribution between patients with primary and recurrent OPC (*P *= 0.264).

**Table 1:** Distribution of oral yeast isolates among HIV-infected patients with primary and recurrent oropharyngeal candidiasis

**Species**	**Patients with primary OPC**		**Patients with recurrent OPC**		**All patients with primary and recurrent OPC**	
	**n**	**(%)**	**n**	**(%)**	**n**	**(%)**
*Candida albicans*	141	(47.6)	109	(36.9)	250	(84.5)
*Candida glabrata*	8	(2.7)	12	(4.1)	20	(6.8)
*Candida krusei*	4	(1.35)	6	(2.05)	10	(3.4)
*Candida tropicalis*	6	(2.02)	2	(0.68)	8	(2.7)
*Candida kefyr*	3	(1)	-	-	3	(1)
*Saccharomyces cerevisiae*	2	(0.68)	1	(0.3)	3	(1)
*Candida pintolopesii*	-	-	1	(0.3)	1	(0.3)
*Candida dubliniensis*	1	(0.3)	-	-	1	(0.3)

**Total of all isolates**	**165**	**(55.7)**	**131**	**(44.3)**	**296**	**(100)**

### *In vitro *susceptibility of isolates

The results of antifungal susceptibility, as summarized in Table 2, show that only fifteen of the 296 isolates (5%) were resistant to fluconazole, while 20 (6.8%) isolates were susceptible dose dependent (SDD) and 261 (88.2%) of all tested isolates were susceptible to fluconazole. Twenty-five (8.4%) isolates were resistant to itraconazole, while 26 (8.8%) were SDD and 245 (82.8%) isolates were susceptible to itraconazole. Fluconazole exhibited greatest activity against *C. albicans *with no resistant strain found; overall five *C. albicans *isolates (2%) were SDD, while 245 (98%) were susceptible to this agent. All *C. krusei *isolates were resistant to fluconazole while 4 of the 8 (50%) *C. tropicalis *isolates were resistant to fluconazole. Only one of the isolated *C. glabrata *isolates (5%) was resistant to fluconazole, while six (30%) were fully susceptible and 13 (65%) were SDD. Moreover, all isolated *C. kefyr*, *S. cerevisiae *and *C. dubliniensis *isolates were fully susceptible to fluconazole, and the only isolated *C. pintolopesii *was SDD. Ten (4%) of the isolated *C. albicans *were resistant to itraconazole, while seven (2.8%) isolates were SDD and 233 (93.2%) isolates were susceptible to this antifungal agent. None of the *C. glabrata *isolated was fully susceptible to itraconazole, but 9 (45%) were SDD and 11 (55%) resistant. None of *C. krusei *isolates was resistant to itraconazole, while 5 (50%) isolates were SDD. *C. pintolopesii *was resistant while *C. dubliniensis *was fully susceptible to itraconazole. All the three *S. cerevisiae *isolates were SDD to itraconazole.

**Table 2:** In vitro antifungal susceptibility of all oral yeast isolates (n = 296)

**Species/Antifungal agent**	**MIC (*μ*/ml)**				%R*
	**MIC Range**	**MIC**_50_	**MIC**_90_	**GM**	
*C. albicans *(250)					
Amphotericin B	0.125 – 1	0.5	0.5	0.326	
Nystatin	2 – >16	4	4	3.377	
Fluconazole	0.063 – 32	0.25	2	0.330	0
Itraconazole	0.016 – >16	0.016	0.063	0.021	4
Miconazole	0.016 – 4	0.016	0.063	0.025	
Clotrimazole	0.125 – 2	1	2	0.474	
					
*C. glabrata *(20)					
Amphotericin B	0.5 – 1	0.5	1	0.545	
Nystatin	2 – 4	4	4	3.668	
Fluconazole	2 – 64	16	32	13.22	5
Itraconazole	0.016 – 4	1	4	0.615	55
Miconazole	0.063 – 0.5	0.25	0.5	0.189	
Clotrimazole	0.125 – 2	1	2	0.474	
					
*C. krusei *(10)					
Amphotericin B	0.5 – 1	1	1	0.683	
Nystatin	2 – 8	4	4	0.965	
Fluconazole	64 – >64	64	>64	76.1	100
Itraconazole	0.031 – 0.5	0.125	0.5	0.134	0
Miconazole	1 – 2	2	2	1.464	
Clotrimazole	0.063 – 0.25	0.125	0.125	0.116	
					
*C. tropicalis *(8)					
Amphotericin B	0.25 – 1	0.5	-	0.458	
Nystatin	2 – 4	4	-	3.084	
Fluconazole	1 – >64	16	-	14.67	50
Itraconazole	0.016 – 2	0.5	-	0.176	37.5
Miconazole	0.125 – 4	2	-	0.840	
Clotrimazole	0.031 – 4	2	-	0.337	
					
*C. kefyr *(3)					
Amphotericin B	0.5 – 0.5	0.5	-	0.5	
Nystatin	2 – 4	2	-	2.244	
Fluconazole	0.25 – 0.5	0.5	-	0.396	0
Itraconazole	0.016 – 0.031	0.031	-	0.019	0
Miconazole	0.016 – 0.016	0.016	-	0.016	
Clotrimazole	0.016 – 0.016	0.016	-	0.016	
					
*C. pintolopesii *(1)					
Amphotericin B	0.5	-	-	-	
Nystatin	4	-	-	-	
Fluconazole	16	-	-	-	0
Itraconazole	1	-	-	-	100
Miconazole	0.25	-	-	-	-
Clotrimazole	1	-	-	-	-
					
*C. dubliniensis *(1)					
Amphotericin B	0.25	-	-	-	
Nystatin	4	-	-	-	
Fluconazole	0.5	-	-	-	0
Itraconazole	0.016	-	-	-	0
Miconazole	0.016	-	-	-	
Clotrimazole	0.016	-	-	-	
					
*S. cerevisiae *(3)					
Amphotericin B	0.5 – 1	0.5	-	0.445	
Nystatin	2 – 4	4	-	2.828	
Fluconazole	8 – 8	8	-	8	0
Itraconazole	0.25 – 0.5	0.25	-	0.353	0
Miconazole	0.063 – 0.25	0.063	-	0.088	
Clotrimazole	0.063 – 1	0.063	-	0.099	
					
All isolates (296)					
Amphotericin B	0.125 – 1	0.5	0.5	0.352	
Nystatin	2 – >16	4	4	3.39	
Fluconazole	0.063 – >64	0.5	16	0.592	5
Itraconazole	0.016 – >16	0.016	0.5	0.031	8.4
Miconazole	0.016 – 4	0.031	1	0.036	
Clotrimazole	0.016 – 4	0.016	1	0.029	

MIC_50 _– MIC value able to inhibit 50% of the isolates tested.

MIC_90 _– MIC value able to inhibit 90% of the sample tested.

GM – Geometric Mean.

R* – Percent resistance using interpretive breakpoint criteria of the CLSI (2002): Itraconazole resistance ≥ 1; Fluconazole resistance ≥ 64 *μ*g/ml.

### Correlation between MIC and recurrence of OPC

In this study, among 292 patients investigated 173 patients (59.2%) had a prior history of antifungal usage, while 119 patients (40.8%) were antifungal treatment naïve. Therefore, 176 isolates (59.5%) were isolated from patients who had previous exposure to antifungal therapy (Table 3). Among patients exposed to antifungal therapy, 94 (54.3%) were exposed once, 47 (27.2%) twice and 32 (18.5%) were exposed three times and above. All patients with recurrent OPC had exposure to antifungal agents before enrollment in our study. Regarding patients with primary OPC, 45 (27.4%) were exposed to antifungal agents due to other fungal infections including vaginal candidiasis, skin fungal infections and cryptococcal meningitis. Most of patients had exposure to more than one antifungal agent, including fluconazole, clotrimazole, nystatin, miconazole and ketoconazole. Among exposed patients, 149 patients (86.1%) were exposed to azole compounds with fluconazole being the predominant (49.7%).

**Table 3:** *In vitro* susceptibility of oral isolates to fluconazole and itraconazole as related to previous antifungal therapy

**Antifungal**	**Previous antifungal (n = 176^a^)**			**No previous antifungal (n = 128^b^)**			***P*-value**
	**S**	**SDD**	**R**	**S**	**SDD**	**R**	
Fluconazole	149 (84.7)	17 (9.7)	10 (5.7)	112 (93.3)	3 (2.5)	5 (4.2)	0.037
Itraconazole	133 (75.6)	22 (12.5)	21 (11.9)	112 (93.3)	4 (3.3)	4 (3.3)	0.002

Data are expressed as number of isolates (percentage).

S – Susceptible, SDD – Susceptible dose dependent, R – resistant.

Interpretive breakpoint criteria of the CLSI (2002): Fluconazole; S (MIC ≤ 8 *μ*g/ml), SDD (MIC 16 – 32 *μ*g/ml), R (MIC 64 *μ*g/ml) and Itraconazole; S (MIC ≤ 0.125 *μ*g/ml), SDD (MIC 0.25 – 0.5 *μ*g/ml), R (MIC ≥ 1).

^a^*C. albicans *(143), *C. glabrata *(19), *C. tropicalis *(4), *S. cerevisiae *(2), *C. pintolopesii *(1).

^b^*C. albicans *(107), *C. glabrata *(1), *C. tropicalis *(4), *C. kefyr *(3), *S. cerevisiae *(1), *C. dubliniensis *(1).

There were significant differences in the MIC distributions for isolates from patients with primary and recurrent OPC for amphotericin B (*P *= 0.041), itraconazole (*P *= 0.008), miconazole (*P *= 0.045), and clotrimazole (*P *= 0.013). No significant difference in MIC distribution between isolates was observed for nystatin (*P *= 0.608) and fluconazole (*P *= 0.133). Based on CLSI interpretative breakpoints, fluconazole resistance for species isolated from patients with primary OPC and recurrent OPC was noted in 9 isolates (5.5%) and 6 isolates (4.6%), respectively, which was not different (*P *= 0.581). However for itraconazole, more isolates cultured from patients with recurrent OPC exhibited reduced susceptibility compared with those cultured from patients with primary OPC (*P *= 0.03).

However, when the correlation between in vitro susceptibility and previous exposure to antifungal agents was determined, it was found that all azole compounds tested were less active against *Candida *isolates cultured from patients who had been previously exposed to any antifungal drug compared with those cultured from the treatment naïve group (fluconazole, *P *= 0.037; itraconazole, *P *= 0.002; miconazole, *P *= 0.015; clotrimazole, *P *= 0.007). No significant differences in MICs of amphotericin B and nystatin between the two groups of isolates were noted (*P *= 0.087 and *P *= 0.117), respectively.

In the present study, two of the five *C. albicans *isolates that were SDD to fluconazole were also SDD to itraconazole and three isolates were resistant to itraconazole. Itraconazole resistance was in 25 (8.4%) of all tested isolates, among which 17 isolates (68%) had MICs of ≥ 1 *μ*g/ml for clotrimazole.

## Discussion

The present study demonstrates that in Tanzania primary and recurrent OPC is predominantly caused by *C. albicans*. There was no significant difference in species distribution between the two groups of patients (*P *= 0.264). There are only a limited number of studies done in African countries regarding distribution of oral yeasts in HIV-infected patients. However, similar to our results, studies done in South Africa demonstrated that more than 80% of oral yeast isolates from HIV-infected patients were of the species *C. albicans *[23,31,32] that is not different to reports from other continents [19,33,34]. On looking at distribution of isolates from patients with recurrent OPC and previously exposed to antifungal therapy, the current results are contrary to what was expected. Previously studies suggested that, repeated exposure to antifungal agents and recurrent infections might predispose to a shift to non-albicans *Candida *species [19,20]. This was not the case in this study, which could be explained by the fact that, majority of the patients with recurrent OPC had only one previous episode of OPC and among patients previously exposed to antifungal therapy almost a half were exposed only once. Interestingly, the first *C. dubliniensis *has been isolated from a Tanzanian HIV/AIDS patient with primary OPC and without previous history of antifungal therapy. Previous studies, reported that *C. dubliniensis was *primarily isolated from patients with previous antifungal therapy and/or recurrent OPC [35-37].

In the present study, there appears to be a significant rapid induction of reduced susceptibility to azoles among oral yeasts isolated from patients with recurrent OPC and a previous history of antifungal therapy. This suggests that patients with more recurrent episodes of OPC and repeatedly exposed to antifungal therapies pose a great risk of reduced susceptibility to azole antifungal agents. Similarly, previous studies reported that prolonged exposure to antifungal therapy of these patients lead to reduced susceptibility of *Candida *species [17,19,38,39]. *Candida *resistance to azole compounds has frequently been attributed to a selective pressure caused by antifungal agents and azole cumulative doses due to exposure to several courses of short- or long-term suppressive therapies in patients with OPC [38-40]. Similarly, the risk of developing OPC with reduced susceptibility to azoles has been associated with greater duration of HIV infection and severe immunosuppression [20].

Generally, even with the observed reduced susceptibility to azoles among oral yeast isolated from patients with recurrent OPC and previous history of antifungal therapy, in the present study resistance to antifungal agents among the tested isolates is relatively low. Resistance was mainly among non-albicans *Candida *species that are primarily or intrinsically resistant to azoles [19]. Fluconazole resistance among all the tested isolates was 5%, which is similar to that reported by other previous studies [21,41]. The reason for the low fluconazole resistance could be explained by the fact that, fluconazole was non affordable to most HIV patients until October 2004, when the government started to provide it for free to needy HIV-infected patients as standard of care. In general, antifungal drug prophylaxis is rarely practiced in Tanzania. In the present study, overall resistance of all tested isolates to itraconazole is 8.4%; slightly higher than that of fluconazole (5%). However, none of the investigated patients were exposed to this antifungal agent before, indicating possible cross-resistance following exposure to other compounds from this class [42,43]. A relation between azole resistance and previous azole exposure was found in this study, with patients who were previously exposed to antifungal therapy having isolates significantly more resistant to itraconazole and fluconazole than those from treatment naïve patients (*P *= 0.002) and (*P *= 0.037), respectively.

There are no interpretative breakpoint criteria for nystatin, amphotericin B, miconazole and clotrimazole. However, the results of the present study have shown that MICs of miconazole, clotrimazole and amphotericin B was concentrated in a narrow range. Previous studies have shown low MICs of miconazole and amphotericin B against tested strains [17,21,24], which is in accordance with our findings. The Tanzanian guidelines for management of HIV and AIDS include fluconazole orally, miconazole, nystatin oral suspension, 2% Sodium benzoate solution or gentian violet solution and ketoconazole for oropharyngeal and esophageal candidiasis [44]. Amphotericin B is used in topical forms as an oral suspension, but the drug is not available for this indication in Tanzania The present study would support the effectiveness of amphotericin B, miconazole, clotrimazole, itraconazole and fluconazole for treatment of OPC in HIV/AIDS patients. However, continuously surveillance programs are needed in order to identify possible changes in the species distribution, antifungal susceptibility patterns of oral yeasts and in vivo efficacy of these antifungal agents.

## Conclusion

This study demonstrates that *C. albicans *is the most frequently isolated species from both patients with primary and recurrent OPC. Oral yeast isolates from Tanzania have high level susceptibility to fluconazole, itraconazole, miconazole, clotrimazole and amphotericin B. Recurrent oropharyngeal candidiasis and previous antifungal therapy significantly correlated with reduced susceptibility to fluconazole, itraconazole, miconazole, and clotrimazole.

## Authors' contributions

All authors participated in the design, implementation, analysis, and interpretation of the study and commented on the draft of the report. All authors read and approved the final manuscript.
